# Design of a prospective cohort study to assess ethnic inequalities in patient safety in hospital care using mixed methods

**DOI:** 10.1186/1472-6963-12-450

**Published:** 2012-12-07

**Authors:** Floor van Rosse, Martine C de Bruijne, Cordula Wagner, Karien Stronks, Marie-Louise Essink-Bot

**Affiliations:** 1 Department of Public Health, Academic Medical Center, Meibergdreef 9, Amsterdam, AZ 1105, The Netherlands; 2 Department of Public and Occupational Health, VU University Medical Center (VUmc), EMGO Institute for Health and Care Research, Amsterdam, The Netherlands; 3 NIVEL, Netherlands Institute for Health Services Research, Utrecht, The Netherlands

## Abstract

**Background:**

While US studies show a higher risk of adverse events (AEs) for ethnic minorities in hospital care, in Europe ethnic inequalities in patient safety have never been analysed. Based on existing literature and exploratory research, our research group developed a conceptual model and empirical study to increase our understanding of the role ethnicity plays in patient safety. Our study is designed to (1) assess the risk of AEs for hospitalised patients of non-Western ethnic origin in comparison to ethnic Dutch patients; (2) analyse what patient-related determinants affect the risk of AEs; (3) explore the mechanisms of patient-provider interactions that may increase the risk of AEs; and (4) explore possible strategies to prevent inequalities in patient safety.

**Methods:**

We are conducting a prospective mixed methods cohort study in four Dutch hospitals, which began in 2010 and is running until 2013. 2000 patients (1000 ethnic Dutch and 1000 of non-Western ethnic origin, ranging in age from 45-75 years) are included. Survey data are collected to capture patients’ explanatory variables (e.g., Dutch language proficiency, health literacy, socio-economic status (SES)-indicators, and religion) during hospital admission. After discharge, a two-stage medical record review using a standardized instrument is conducted by experienced reviewers to determine the incidence of AEs. Data will be analysed using multilevel multivariable logistic regression. Qualitative interviews with providers and patients will provide insight into the mechanisms of AEs and potential prevention strategies.

**Conclusion:**

This study uses a robust study plan to quantify the risk difference of AEs between ethnic minority and Dutch patients in hospital care. In addition we are developing an in-depth description of the mechanisms of excess risk for some groups compared to others, while identifying opportunities for more equitable distributions of patient safety for all.

## Background

Patient safety, defined as the lack of preventable injury, is the minimum prerequisite for good quality of care. Safety should be equally achievable for all patients, independent of their backgrounds. 11% of the population currently living in the Netherlands is considered to be of non-Western origin [see Table 1, and this percentage is increasing, as it is all over Europe. International literature outside Europe shows ethnic inequalities in patient safety [1-12]. To our knowledge, ethnic inequalities in patient safety have not been studied before in any European contexts.

**Table 1:** Ethnic background classification

Dutch ethnic origin	A patient is classified as ‘Dutch’ when the patient and one or both parents of the patient were born in the Netherlands, or when the patient was born outside the Netherlands while both parents were born in the Netherlands.
Non-Western ethnic origin	A patient is classified as ‘non-Western’ when the patient and one or both parents of the patient were born in a non-Western country^a^, or when both parents were born in a non-western country, irrespective of the country of birth of the patient.
Classification of country of origin for non-western patients	When a patient and one or both parents were born in the same country, e.g. Turkey, that country is the country of origin, the patient will be classified as ‘Turkish’. When both parents of the patient were born in the same country, which is different from the country of birth of the patient, the country of birth of both parents is the country of origin. When a patient and his/her parents were born in three different countries, the country of birth of the mother will indicate the country of origin of the patient.
	An exception exists for our ethnic origin definition regarding Javanese Surinamese patients who were born in Suriname and whose both parents were born in Indonesia. According to the CBS definition they would be classified as “western”. But they are included in our non-western study sample and classified as Javanese Surinamese.

^a ^Non-Western countries are: Turkey, all countries in Africa, all countries in South America, and all countries in Asia except Japan and Indonesia.

This paper is laid out in three parts. It starts with an overview of published empirical studies outside of Europe assessing ethnic inequalities in patient safety. We also describe two exploratory studies conducted by our research group. Secondly, we present a conceptual model that has helped us theorize the possible association between ethnicity and patient safety. Finally, we describe the design of our current study that assesses and describes possible ethnic inequalities in patient safety in Dutch hospital care.

### Ethnic inequalities in patient safety – empirical literature

Additional file 1 provides an overview of studies that have examined ethnicity in relation with patient safety in hospital care [1-12]. Those published to date have documented the U.S. and New Zealand’s healthcare systems. Because both of these contexts differ from the Dutch and other European systems, and different definitions of ethnicity are also employed, results are not easily generalised and a study of European inequalities can make an important contribution.

In advance of our current study, our research group performed two exploratory studies about ethnic inequalities in patient safety in the Netherlands. First, we conducted a nationwide retrospective record linkage study to explore whether ethnicity was associated with excess lengths of stay (LOS) and unplanned readmission rates [13]. We determined that, overall, ethnic minority groups had more unplanned readmissions and excess LOS compared to ethnic Dutch after controlling for demographics and patient mix (significant Odds Ratios (ORs) varying from 1.04-1.14). These differences were partly but not largely accounted for by socio-economic indicators, meaning that variations in socio-economic status (SES) explained part but not all of the risk differences between ethnic groups. In interpreting our findings, we determined that excess LOS and unplanned readmission might be distal indicators of AEs, suggesting a differential risk of poor patient safety with ethnic background. The interpretation was inconclusive, however, because the differences could also be explained by each group’s healthcare needs.

Secondly, we conducted a qualitative interview study with care providers, to better understand the process underlying ethnic differences in patient safety [14]. Three patterns of interactions between professional and patient that can contribute to ethnic differences in patient safety were identified: inappropriate responses by health care providers to objective characteristics of immigrant patients, such as low Dutch proficiency; misunderstandings between patients and care providers due to differences in illness perceptions and expectations about health care; and inappropriate care because of providers’ prejudices against or stereotypical ideas about patients of non-Dutch ethnic origin.

### Ethnic differences in patient safety – conceptual model

**Figure 1:**
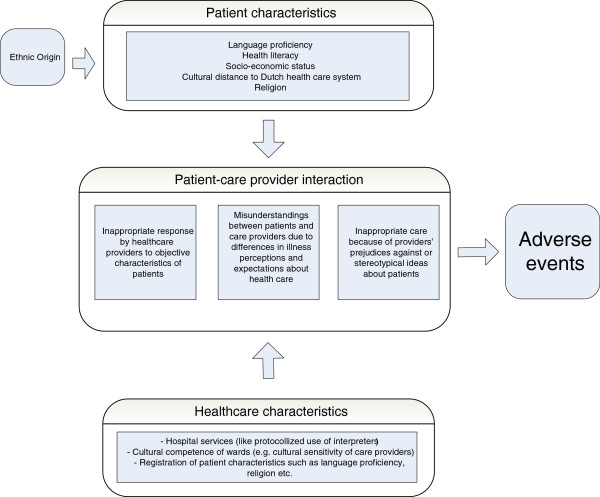
Conceptual model.

Existing literature and our exploratory work suggest the possibility of a higher risk of AEs among ethnic minority patient groups, but valid epidemiological evidence for the situation outside of the U.S. and New Zealand is lacking. Based on the exploratory studies, we developed a conceptual model to understand the role ethnicity may play in patient safety (Figure 1). Our model identifies three domains: 1) Patient characteristics (such as language proficiency): here, we relied on Stonks et al. [15] to define relevant patient characteristics that possibly explain ethnic differences in patient safety. 2) Healthcare characteristics (such as a protocolled use of interpreters): we also relied upon Stronks et al. [15] along with the CLAS standards to define healthcare characteristics [16]. 3) Patient-care provider interaction: we used the work of Suurmond et al. [14].

Besides measuring differences in risk, throughout our study we are collecting data to identify the determinants and mechanisms that potentially increase the risk of AEs for ethnic minority groups.

Study objectives are to:

1) determine and compare incidence, type, impact, and preventability of AEs in non-Western hospital patients compared to ethnic Dutch hospital patients.

2) assess the contribution of patient-related determinants (language proficiency, health literacy, SES indicators, cultural distance to the Dutch healthcare system and religion), and the contribution of healthcare-related determinants to the risk of experiencing an AE in hospital care.

3) explore the mechanisms of patient-provider interactions that may increase the risk of AEs.

4) explore possible strategies to prevent inequalities in patient safety.

## Methods

### Design overview

**Figure 2:**
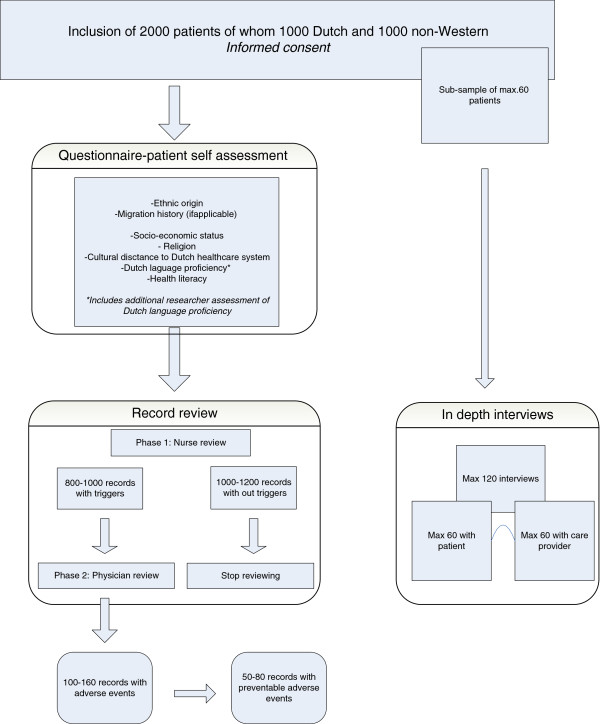
Flow chart of measurements and primary outcome.

We are using a mixed methods design combining quantitative and qualitative methods. Quantitative methods include the use of a survey-based questionnaire and a medical record review to compare the incidence of AEs in Dutch and non-Western patients (See Table 1) and to assess the contribution of several determinants to the risk of AEs. We use a qualitative interview-based component to examine the mechanisms underlying AEs, and to explore possible prevention strategies. Figure 2 provides a schematic overview of the study design and outcome. Data collection began in 2010, and is projected to be completed in 2013.

### Setting

Patient recruitment is taking place in four urban hospitals in three cities in the Netherlands with a high density of inhabitants classified as being of non-Western ethnic origin. Two of the hospitals are teaching hospitals. In each of the hospitals, a total of 500 patients will be included, equally divided between ethnic Dutch and non-Western patients (See Table 1).

### Study population

We are in the process of recruiting all 2000 patients, of whom approximately half are considered ethnically Dutch, and the other half are considered of non-Western ethnic origin (Table 1). To define Dutch origin and non-Western origin, we are using ethnicity indicators based on country of birth criteria as defined by the Dutch Central Bureau of Statistics [17] (See Table 1). In the Netherlands and elsewhere in continental Europe, ethnic origin based on country of birth is commonly used as the ethnicity indicator. This is distinct from the UK, which uses self-identified ethnicity, and in the U.S., where both ethnicity and race are used to classify groups [18].

Place of birth of patients, and their parents, is not registered in Dutch hospitals in a standard way. This information, along with patient-specific variables (Dutch language proficiency, SES-indicators, religion, health literacy, and cultural distance to the Dutch healthcare system) are obtained in the patient self-assessment questionnaire.

### Background of ethnic minorities in the Netherlands

The three largest groups of residents of non-Western ethnic origin living in the Netherlands are of Turkish, Surinamese, and Moroccan origin. The Turkish and Moroccan groups mainly came to the Netherlands between 1960 and 1980 in the wave of economic labour migration. Migrants generally had a low level of education at the time of entry, which persists today. The Surinamese group migrated from Suriname, a former Dutch colony in South America. The Surinamese population is often divided into two principal subgroups: one of African (Creole) and the other of South Asian (Hindustani) decent. Migration to the Netherlands peaked at the time of Surinamese independence in 1975. Most people of Surinamese origin speak the Dutch language. Those of non-Western ethnic origin that we describe primarily live in the three largest Dutch cities (Amsterdam, Rotterdam, and The Hague) and often cluster in certain neighbourhoods.

### Inclusion and exclusion criteria

We include older patients and clinical admissions rather than day-admissions in our study. Patient safety research shows that incidence of AEs is higher among older patients and among those who undergo invasive or complex care [19]. Also, the 2009 record linkage study showed that ethnic differences in LOS and readmission were larger among older patients [13], and so we therefore include patients between ages 45 and 75 years of age. The lower limit of 45 was chosen because from this age, the hospital admission rate increases. By choosing 45 years as a lower limit, around 98% of the non-Western patients in our study will be first generation immigrants, meaning they were born in their country of origin that designates their ethnic classification. The upper age limit of 75 years was chosen because in the Netherlands, there were few non-Western inhabitants prior to the above-described waves of migration.

For logistical reasons, patient recruitment has been limited to three large wards in each hospital: surgery (including orthopaedic surgery), internal medicine (including cardiology and pulmonology), and neurology. To enhance comparability between ethnic Dutch and non-Western patients regarding type of condition and reason for admittance, we have ensured that we enrol approximately the same number of representatives of each group in each of the wards, i.e., no more than 10 more in one group compared to the other per ward.

Because subjects of non-Western origin in the study’s age group on average score lower in variable categories that potentially increase the risk of AEs (e.g., Dutch language proficiency and health literacy) compared to people of Western but non-Dutch ethnic origin, we have selected to exclude the latter group. By doing so, the contrast between the two study groups in differential risk of AEs should be increased (See Table 1).

### Sample size

A sample size of 1000 patients per group allows us to detect an incidence difference of 2% by an incidence of 8%, for which we need at least 800 patients per group. In order to create enough power to investigate the association between the five explanatory patient-related variables (See Figure 1) and an AE, we need 200 extra patients per group. The sample size calculation is based on those from prior record review studies to measure differences in AE incidences per hospital type, and based on the results of the most recent Dutch record review study [20,21].

### Recruitment

#### Ward recruitment

We informed wards through an oral presentation and an information letter before the study started. Only limited efforts have been required of ward staff. On recruitment days, we ask the co-operation of a senior nurse to identify which patients selected by the researchers can be approached and have no severe medical contraindications (See ‘Patient recruitment’). We have asked the ward management to provide basic ward characteristics (see ’Measurements-healthcare level’). A limited number of care providers are invited for an interview. (See ‘Qualitative component’).

### Patient recruitment

Patients are recruited on the wards during their clinical admissions. To reach recruitment targets, each ward is visited once or twice a week, depending on patient turnover. At each visit, the presence of patients meeting the inclusion criteria is assessed by the researchers using the wards’ admissions information. Then a senior nurse verifies whether these eligible patients are available to be approached by the researcher or her assistants. The senior nurse may only advise against approaching prospective patients for serious reasons, defined by the medical condition or the patient being busy with or recovering from a test or intervention. If possible, these patients are approached on a subsequent visit, which is most of the times successful.

At each ward visit, we approach the latest admitted eligible Dutch and non-Western patient, followed by the second to last admitted eligible Dutch and non-Western patient, and so on as a way of avoiding selection bias. Before a patient is approached, his/her ethnic background is estimated by the researcher based on surname, phenotypical characteristics, and the presumptions of a senior nurse. Ethnic origin is subsequently confirmed by patient through the self-assessment part in the questionnaire, which asks the patient about country of birth as well as his/her parents. To ensure not missing any eligible patients, researchers use an approach that initially includes patients who may ultimately be excluded.

An information letter as well as oral information are provided when the researcher approaches the patient. Patients willing to participate must sign an informed consent form. By signing the form, the patient gives researchers permission to review the patient record after discharge, and to use the data from the questionnaire. Informed consent can also be given orally, in which case the consent is taped. After the consent process, the questionnaire is given. Patients who are able to do so fill out the questionnaire independently. When necessary, the researcher helps the patients by reading out the questions and/or writing down the answers. Information letters, consent forms, and questionnaires are available in Dutch, English and Turkish. We have no written translation in Arabic, because we anticipated that many Arabic-speaking patients would not read or write in Arabic. Further, the Berber dialect spoken by Moroccan patients is not a written language. We use trained bilingual (Turkish and Arabic speaking) research assistants to ensure the participation of those who do not speak Dutch or English. A limited number of patients may be invited to participate in an interview after their discharge. (See ‘Qualitative component’).

### Non-response or missing

Throughout, we monitor the number of patients who we could not approach due to the advice of the senior nurse, and, if applicable, the reason for advising against approaching. We are also monitoring the number of patients who refuse to participate, and, when possible, the reason for refusal (e.g., not comfortable with record review, already participating in other studies, feeling too sick to fill out a questionnaire). Finally, we monitor the number of patients we could not approach at all because they were off-ward for a scan or other procedure.

#### Data collection

The data sources for the first two study objectives include patient questionnaires and patient records.

### Patient questionnaire

The questionnaire first captures the demographic characteristics of age, ethnic origin, and (if applicable) migration history.

– Ethnic origin is assessed by country of birth of the patient and birth of the patient’s parents [17]. Additionally, patients of Surinamese ethnic origin are asked to classify to which subgroup they belong (e.g. Hindustani or Creole) [18].

– The two variables on migration history are length of time of residence in the host country (measured by year of arrival in the Netherlands), and reason for migration (patients can choose between: work/study, family, political, or ‘other’).

The questionnaire also contains items about all of the patient-related determinants shown in the conceptual model (See Figure 1).

– Dutch language proficiency is measured by asking patients to rank their own proficiency in understanding, speaking, reading, and writing the Dutch language (classified as ‘not at all’, ‘a little’, ‘moderately’, ‘good’), and by asking which language is spoken at home.

– Religion is measured by asking whether patients are religious, and if so, which religion they practise/were brought up with. Also, we assess whether patients practise their religion by asking if they visit religious services and how often.

– SES-indicators are measured with items about education, occupation, and income. Educational level is measured by asking about the number of completed years of education since six years of age and the highest completed grade level. We use reply-assistance reference cards depicting educational systems of the Netherlands, Suriname, Turkey, and Morocco. Occupation is measured by asking the patient’s current employment status, and, if applicable the employment status of his/her partner. Because of privacy reasons, we will not ask for income amounts, but rather if and how easily patients are able to run their household based on their income.

– Health literacy is measured with three items using Chew’s Set of Brief Screening Questions (SBSQ). The questions are: "How often do you have someone help you read hospital materials?"; "How confident are you filling out medical forms by yourself?"; and "How often do you have problems learning about your medical condition because of difficulty understanding written information?" The combined item-responses result in a subjective health literacy score [22-24]. We use the Dutch adaptation of the SBSQ [25].

– Cultural distance to the Dutch healthcare system is measured with five newly developed items in which patients are asked to compare the Dutch healthcare system with the healthcare system in the country of their ethnic origin. When applicable, patients are asked to indicate which system they prefer.

Questions on migration history and cultural distance to the Dutch healthcare system are only provided to non-Western patients.

Because we approach patients during hospital admission, a time when they may feel ill, we made the questionnaire as short as possible to lessen the burden of participation as much as possible.

### Researcher assessment

In addition to the patient-self assessment, the researcher reports his or her perception of the patient’s Dutch language proficiency and health literacy for all enrolled patients. All communication problems due to language barriers, the patient’s medical condition, or other factors are reported. Furthermore, because phenotypical characteristics such as skin colour may be related to stereotyping in healthcare, these are also reported by the researcher.

Data entry of questionnaires is performed in Blaise®. During data collection, data checks are performed on a regular basis (e.g. inconsistencies, missing data, out of range answers).

### Measurements – healthcare level

At the hospital level, we collect characteristics such as the number of beds, admissions per year, and the type (i.e., teaching hospital). Furthermore we investigate the different hospital-specific services that are available (e.g., interpreters, spiritual counselors, dietary services).

At ward level, characteristics and cultural competence data are collected including basic information about employees (e.g., distribution of age, sex, and ethnic origin), number of beds, and patients’ average lengths of stay. Cultural competence data include the use of interpreters, special meals, and spiritual counselors in the ward.

### Record review

After four months after the patients discharge, the medical record is screened for the presence of AEs in a two-stage review process based on the Harvard Medical Practise Study (HMPS) [26] and Dutch patient safety studies [20,21]. In the first stage, nurses review the complete nursing record for the presence of one or more of 16 triggers known to be sensitive to the presence of an AE (See Table 2). If one or more triggers are found in the nursing record, the record is forwarded to the second stage of the review procedure. When no triggers are found in the nursing record, the nurse screens the medical record for the presence of triggers. When triggers are present, the nurse decides the specialty most appropriate to review the record in the second phase, choosing between internal disease, surgery, or neurology.

**Table 2:** Triggers

1	Unplanned admission before index admission (admission reasons are related to the index admission)
2	Unplanned readmission after discharge
3	Hospital-incurred patient injury (Permanent or temporary injury obtained (acquired) during index admission)
4	Adverse drug reaction
5	Unplanned transfer from general care to (an) intensive care (unit)
6	Unplanned transfer to another acute care hospital (after unexpected deterioration of the patient)
7	Unplanned return to the operating room
8	Unplanned removal, injury, or repair of organ during surgery
9	Hospital-acquired infection or sepsis (initiated >72 hours after admission)
10	Other patient complication
11	Development of neurological deficit not present on admission
12	Unexpected death
13	Cardiac or respiratory arrest
14	Inappropriate discharge to home
15	Dissatisfaction with care documented in the medical record
16	Any other undesirable outcome not covered above

### Adverse event assessment

The specialist determines whether an AE has occurred, and whether it was preventable.

The determination of AEs is based on three criteria [20] (See Table 3). First, the specialist determines if unintended injury occurred. A 6-point scale is used to determine whether the injury was caused by healthcare management rather than by the patients’ disease. To structure the review process, the causation scale is preceded by 13 questions to facilitate the final judgement (See Additional file 2). Causation scores 4-6 are classified as AEs and further analysed. In addition, the degree of preventability (6-point scale) is determined, again preceded by 13 facilitating questions. Furthermore, timing, involved specialties, parts of the care process (e.g., diagnosis, medication, discharge) and causes (e.g. technical, human factors) of AEs are determined [20,21].

**Table 3:** Adverse event definition


Adverse event	An unintended injury^a ^that results in disability that results in temporary or permanent disability, death, or prolonged hospital stay and is caused by health care management rather than by the patient’s underlying disease process.
	In our study, determination of the presence of an adverse event was based on 3 criteria:
	1. An unintended (physical and/or mental) injury which
	2. Results in temporary or permanent disability, death or prolongation of hospital stay, and is
	3. caused by health care management rather than the patient’s disease
Preventable adverse event	An adverse event resulting from an error in management due to failure to follow accepted practice at an individual or system level. Accepted practice is the ‘current level of expected performance for the average practitioner or system that manages the condition in question’.

^a ^Any disadvantage for the patient that leads to prolonged or strengthened treatment, temporary or permanent (physical or mental) impairment or death.

### Characteristics of the hospital admission

To describe the patient mix, the following variables are also collected from the record: length of stay, admission status (e.g., elective, urgent), admission and discharge diagnosis, admission specialty, and discharge status (e.g., to home, to home with outpatient care). We collect ICD-9-CM codes of the primary diagnosis of all patient admissions.

### Specific patient characteristics

Additionally, to measure registration of patient-related characteristics, the patient records will be searched for entries on:

– the patient’s language proficiency

– communication problems (including communication problems due to the patient’s medical situation)

– the use of interpreters

– the patient’s religion

– the use of special services such as a spiritual counsellor

### Reliability study

To assess the reliability of screening for triggers by nurses, 5% of the records are screened independently by a second nurse. To assess reliability of AE determination, 10% of the second phase records are reviewed independently by a second specialist. The second reviewer is blind to the outcome of the first review.

### Data management

Record review data entry is performed in a highly secured web-based program. During data collection, data checks are performed on a regular basis for inconsistencies, missing data, and out of range answers.

### Reviewer recruitment and training

We recruited experienced reviewers who have participated in other Dutch record review studies [20,21], and who were chosen by using strict selection criteria (at least ten years post graduate general clinical experience for specialists and a minimum of five years of clinical experience for nurses). Reviewers do not review records in hospitals where they are currently working, or where they have worked in the past 20 years.

During data collection, reviewers can discuss problems, and they also use a regularly updated Frequently Asked Questions document. Reflection days are also organised for all reviewers. Reviewers are compensated for their review activities at an hourly rate, and expenses are reimbursed.

#### Qualitative component

To satisfy our third and fourth research objectives, we use qualitative research methods to explore the mechanisms that play a role in AEs in patients of different ethnic origins, and we also examine what prevention strategies might be developed to minimize differences. This quantitative part shows us the contribution of several determinants to the risk of an AE, and examines the relationships between determinants and occurrences of AEs. We are trying to identify the barriers in patient-provider interactions, to find opportunities to improve communication and minimize the risk for AEs.

We are selecting a maximum of 60 admissions by purposive and theoretical sampling based on the admissions where an AE has been determined. We sample from a range of patients with different variables, such as language proficiency, health literacy, etc. For each AE, the patient and care provider are interviewed separately using a semi-structured topic list. The care provider can be either a doctor or a nurse, as long as he/she has been closely involved with the patient’s care process. The interview takes place soon after the patient’s discharge to ensure the patient and provider will remember the details of the admission. In the interview, both patients and providers are asked to explain the AE and the care process in detail. We ask about the perceived barriers that may underlie an AE, and ask interviewees for suggested strategies to address them.

Interviews will be transcribed and qualitatively analysed based on the conceptual model. Transcribed interviews will be coded and text-parts with the same code will be grouped to identify sub-themes. A second researcher will be involved in identifying codes and subthemes.

#### Statistical analysis

Data will be analysed using SPSS 16.0 for Windows or higher.

Baseline comparability will be investigated by descriptive statistics including age, gender, admission status and primary diagnosis of the patients. Also, we will compare some hospital- and ward characteristics such as the number of patients included per ward. Response rates in both groups will be compared. General characteristics of non-responders will be compared to those of responders.

To answer our first research question regarding differences in AE rates between non-Western and ethnic Dutch patients, we will first describe the nature, preventability, causes and consequences of AEs. We will show preventability of AEs per group (both treated as a categorical and dichotomous variable) in order to find out whether differences in the degree of preventability are present. We will tabulate the distribution of AEs across ethnic groups, medical specialties, medical process involved (e.g. diagnosis, medication), impact of AEs, and causes of AEs (e.g. technical, organizational).

Next, incidence rates of AEs and preventable AEs will be calculated for both groups with 95% confidence intervals. Multilevel analysis will be performed to assess and adjust for variance at the hospital and ward level. Potential confounders like age, sex, and primary diagnosis will be entered stepwise into the model.

To answer our second research question, we will perform multilevel multivariable logistic regression analysis to investigate the contribution of the patient-related independent explanatory variables (SES indicators, Dutch language proficiency, health literacy, cultural distance, and religion) to the risk of experiencing an AE. Co-variables will be entered stepwise into the model, starting with potential confounders (age, gender, primary diagnosis), followed by possible explanatory variables. Outcomes will be presented in odds ratios.

To analyse inter-rater reliability, we will calculate the percentage of records for which there was agreement on the presence of screening criteria for nurses and on the presence and preventability for physicians. Also, we will calculate a kappa-statistic.

To prepare for the main analyses, we will conduct exploratory analyses to optimize our dataset and statistical models. To describe our exploratory determinants (Dutch language proficiency, Health literacy, SES-indicators, religion and cultural distance) we have measured several variables. Exploratory analyses will lead us to determine which data to use in further analyses, the cut-off points, and the number of categories the explanatory determinants should be divided into. For example, to measure Dutch language proficiency we have the patient-self assessment of his/her proficiency in understanding, speaking, reading, and writing Dutch, all on a 4-point scale (from ‘not at all’ to ‘good’). Furthermore, we know the language the patient speaks at home, and we also have information on language proficiency from the patient record and the subjective researcher assessment of the patient’s Dutch language proficiency. After exploratory analyses we can decide cut-off points and the number of language proficiency categories, and we can validate our categorization with the researcher assessment. We will use the same method for the health literacy determinant. We also have three SES-indicators (education, occupation, and a proxy for income), and we will choose the optimal ones by refining the SES-measure after exploratory analysis.

## Discussion

This will be the first study to investigate possible ethnic inequalities in Europe. The results of this study will show whether, to what extent, and how ethnic inequalities affect patient safety in Dutch hospital care. Findings will show which determinants and mechanisms potentially increase the risk for an AE, and which of these can be associated with a potential increased risk for ethnic minority groups. With this information, prevention strategies can be formulated, and health care can be made safer for ethnic minorities, and also for all patient groups.

In this study we focus on different explanatory variables that are often applicable to ethnic minority patients rather than analyzing patient safety in specific ethnic groups living in the Netherlands. By identifying specific patient related factors related to AEs, the results will be generalizable to all patients, irrespective of their specific ethnic origin.

### Strengths and limitations

We have made efforts to involve all patients in our study even though they may not have mastered the Dutch language, or be literate. We provide translations of letters, consent forms and questionnaires, use bilingual research assistants, and also ask relatives of patients to interpret when possible. Even with all these efforts, we might miss some patients because they speak a language we are not able to cover. To quantify this possible selection bias, we are closely monitoring how many patients we are unable to approach because of communication difficulties.

Record review has been cited as a strong method to study the frequency and types of AEs, and has high face validity among health care workers. The most important advantages of this method are its utilization of readily available data and its common international use. Other ways of studying AEs are possible, but have important limitations; morbidity and mortality conferences analysis, malpractice claims analysis, and error reporting system analysis are three methods prone to reporting bias, while observation of patient care and clinical surveillance are very expensive and not suitable for detecting latent errors [27]. A weakness of record review is hindsight bias [28]. Knowing the outcome and its severity may influence judgement of causation and preventability. However, this will affect both the groups equally, which means that comparability between them is unaffected. Because we compare two patient groups, assessment bias is another potential limitation. Unfortunately, it is impossible to completely blind reviewers for the patient groups to which the records they review belong. However, this assessment bias can go in both directions. Both over- and under-identification of AEs can occur. Also, because of the standardized format and the number of support questions helping the reviewer make their determinations, we think the risk for this assessment bias is low.

Even with the carefully structured review process and the experience of the reviewers, variation in the judgements of reviewers can be present. We limit variation in judgements by organising reflection days and using frequently updated FAQ-lists. In this study we will have 5% of the first phase and 10% of the second phase records double-reviewed to be able to check inter-reviewer agreement.

To study causes and mechanisms underlying AEs, record review does not provide complete information [27]. In the present study, patient characteristics and patient-provider interaction are very important. The addition of questionnaires and in-depth interviews to our record review constitutes a major strength of this study’s approach.

## Conclusion

This study uses a robust study plan to quantify the risk difference of AEs between ethnic minority and Dutch patients in hospital care. In addition we are developing an in-depth description of the mechanisms of excess risk for some groups compared to others, while identifying opportunities for more equitable distributions of patient safety for all.

### Ethical approval

The study protocol was reviewed and approved by the ethical review board of the Academic Medical Centre in Amsterdam, the Netherlands. All participating hospitals granted approval to participate.

## Competing interests

The authors declare that they have no competing interests.

## Authors’ contributions

FvR, MCdeB and MLE-B designed the study. FvR drafted the manuscript and will collect and analyze the data. MCdB and MLE-B provide daily supervision of the study and the manuscript. KS and CW supervised study design and provided comments on subsequent versions of the manuscript. All authors read and approved the final manuscript.

## Pre-publication history

The pre-publication history for this paper can be accessed here:

http://www.biomedcentral.com/1472-6963/12/450/prepub

## Supplementary Material

Additional file 1 Overview of studies analysing ethnic inequalities in patient safety. Click here for file

Additional file 2 Questions to facilitate the final reviewers’ judgment of causation and preventability. Click here for file
