# Supplementary material for: Design of a prospective cohort study to assess ethnic inequalities in patient safety in hospital care using mixed methods
Source: BMC Health Serv Res. 2012 Dec 7;12:450. doi: 10.1186/1472-6963-12-450 (PMC3570405; doi:10.1186/1472-6963-12-450)
Supplement: Additional file 1 — Overview of studies analysing ethnic inequalities in patient safety. [file 1472-6963-12-450-S1.doc]

**Appendix 1. Overview of studies analysing ethnic inequalities in patient safety**

| **Author, pub year, country** | **Population / Used data** | **Study groups compared** | **Method** | **Outcome measure (definition)** | **Conclusion** |
| --- | --- | --- | --- | --- | --- |
| **Individual quantitative studies** |  |  |  |  |  |
| Miller, 2003, USA | Hospitalised children <19 years  HCUP* data | 1. Black ethnicity  2. Hispanic ethnicity  3. Other ethnicity | Applying PSI algorithms (Records with PSIs vs. without) | AHRQ PSIs** | Black ethnicity is directly associated with birth trauma (OR 1.5) |
| Romano, 2003, USA | Adults and children  HCUP* data | 1. Whites  2. Hispanics  3. African Americans  4. Other/Unknown | Analysis of 20 PSIs | AHRQ PSIs** | African Americans: Higher risk of most medical and nursing-related post operative complications  White patients: slightly higher risk of certain iatrogenic injuries related to procedures. |
| Cohen, 2005, USA | Hospitalised Pediatric patients < 21 | 1. Patients whose families do have language barriers  2. Patients whose families do not have language barriers (interpreter requested = language barrier) | Case-control design, exposure = language barrier | Serious medical events. (Events that led to unintended or potentially adverse outcomes identified by the hospital's quality improvement staff) | Spanish-speaking patients whose families have a language barrier have a significantly increased risk for serious medical events during pediatric hospitalisation compared with patients whose families do not have a language barrier. (OR 2.26) |
| Coffey, 2005, USA | HCUP* data | 1. non-Hispanic blacks  2. non-Hispanic whites  3. Hispanics  4. Asian/Pacific Islanders (APIs) | Analysis of PSIs | AHRQ PSIs** | Higher patient safety event rates for minorities |
| Davis, 2006, New Zealand | Admissions to 13 hospitals in new-Zealand | 1. Maori-patients  2. Patients of non-Maoir/non-pacific origin | Cross-sectional 2 stage record review | Occurrence, effect, and preventability of adverse events | Maori patients have a higher risk of preventable adverse events (adjusted OR: 1.47) |
| Divi, 2007, USA | Adverse event incident reports from 7 hospitals | 1. English speaking patients  2. non-English speaking patients | Classification with Patient Safety Event Taxonomy | Harm | Language barriers appear to increase the risks to patient safety. (49.1 % harm in LEP patients vs 29.5% harm in English patients) |
| Chang, 2008, USA | Trauma patients aged ≥ 18 | 1. Black patients  2. White patients | Retrospective analysis of PSIs | AHRQ PSIs ** | A 20% higher incidence of AEs in black trauma patients compared to white trauma patients |
| Gaskin, 2008, USA | HCUP* data | 1. Whites  2. Blacks  3. Hispanic  4. Asian | Analysis of PSIs | AHRQ PSIs** | Racial disparities in overall adverse event rates. But attributable to quality of hospital |
| Metersky, 2011, USA | Randomly selected charts from hospital discharges | 1. non-Hispanic whites  2. Blacks | Chart abstraction | patient safety events | Hospitalized blacks are at higher risk than whites of experiencing certain patient safety events: infections and adverse drug events (OR 1.34 and 1.29) |
| **Systematic review** |  |  |  |  |  |
| Flores, 2006, USA | pediatric and adult patients | White (100% of studies) African American (100% of studies)  Latino (56% of studies)  Asian/Pacific Islander (22% of studies)  Native American (11% of studies)  Other (78% of studies)  Unknown (11% of studies) | Systematic review of publications | Used methods vary per study. Meta-analysis was not possible | Several racial/ethnic disparities in pediatric patient safety, including higher rates of newborn birth trauma, infections attributable to medical care, and postoperative adverse medical events for minority children, and a greater likelihood of adverse events for hospitalised children whose parents requested a Spanish interpreter (No meta-analysis performed) |

* HCUP: An American Nationwide inpatient sample

**AHRQ PSIs: A set of patient safety indicators developed by the Agency for Healthcare Research and Quality

Qualitative studies

| **Author, pub year, country** | **Findings** |
| --- | --- |
| Johnstone, 2006, Australia | Hypothesis: Language problems and cultural differences increase the risk for adverse events in ethnic minority populations. |
| Mattox, 2010, USA | Paper focusing on nurses, describes several cases, on which author hypothesizes that patients with low English proficiency or inadequate health literacy, and racial and ethnic minority patients are at increased risk for medical error. |

*Search strategy*

We explored the literature by searches related to patient safety (i.e. adverse events, medical error) in hospital care in patients of different ethnic origin. Also studies comparing different language groups were included. We did not include studies assessing patient safety in specific domains (I.e. surgical events, cancer) and we also did not include studies analysing only at hospital level (black-serving hospitals vs. white-serving hospitals). We identified 12 studies of whom nine individual quantitative studies, one systematic review, and two qualitative studies.
