# Supplementary material for: Design of a prospective cohort study to assess ethnic inequalities in patient safety in hospital care using mixed methods
Source: BMC Health Serv Res. 2012 Dec 7;12:450. doi: 10.1186/1472-6963-12-450 (PMC3570405; doi:10.1186/1472-6963-12-450)
Supplement: Additional file 2 — Questions to facilitate the final reviewers’ judgment of causation and preventability. [file 1472-6963-12-450-S2.doc]

Appendix 2**:**

Questions to facilitate the final reviewers’ judgment of causation and preventability.

Causation

- - Is there a note in the medical record indicating that a health carer professional or health care management caused the injury? (No/Yes/Not applicable)
  - Is there a note in the medical record suggesting the possibility of an unintended injury from the patient’s disease? (No/Yes/Not applicable)
  - Does the timing of events suggest that the injury is related to the treatment? (Likely/Possibly/Unlikely/Not applicable)
  - Does the timing of events suggest that the injury was related to the lack of treatment? (Likely/Possibly/Unlikely/Not applicable)
  - Are there other reasonable explanations for the cause of the unintended injury? (No/Yes/Possibly/Not applicable)
  - Was there an opportunity prior to the occurrence of the injury for intervention which might have prevented it? (No/Yes/Possibly/Not applicable)
  - Is lack of treatment or delayed treatment a recognized cause of this injury? (Widely recognized/Recognized by other specialists/No/Not applicable)
  - Is the lack of diagnosis or delayed diagnosis a recognised cause of this injury? (Widely recognized/Recognized by other specialists/No/Not applicable)
  - Is the treatment given to the patient a recognized cause of this injury? (Widely recognized/Recognized by other specialists/No/Not applicable)
  - Is this injury a recognized complication of the patient's underlying index disease? (Widely recognized/Recognized by other specialists/No/Not applicable)
  - Was the injury recognized during the index admission? (No/Yes/Not applicable)
    - *If ‘Yes’* Was the appropriate action taken during the Index Admission? (No/Yes/No action needed/Not applicable)
    - *If ‘Yes’* Did the injury respond to the appropriate action? (No/Yes/Possibly/Not applicable)
  - After consideration of the clinical details of the patient’s management, irrespective of preventability, and your response to the questions above – What level of confidence do you have that the health care professional or health care management caused the injury?
    - 1. (Virtually) no evidence for health care management causation
      2. Slight to modest evidence of health care management causation
      3. Health care management causation not likely (less than 50/50, but 'close call')
      4. Health care management causation more likely (more than 50/50, but 'close call')
      5. Moderate to strong evidence of health care management causation
      6. (Virtually) certain evidence of health care management causation

Preventablity

- - How complex was this case? (Very complex/Moderately complex/Somewhat complex/Not complex/Unable to determine)
  - Was the management of the Primary illness (not the adverse event) appropriate? (Definitely appropriate/Possibly appropriate/Probably appropriate/Definitely not appropriate)
  - What was the degree of deviation of management of the primary illness (not the adverse event) from the accepted norm? (Severe/Moderate/Little/None)
  - What was the co-morbidity of the patient? (Significant co-morbidity/Moderate co-morbidity/Mild co-morbidity/No co-morbidity)
  - What was the degree of emergency in management of the primary illness (not the adverse event) prior to the occurrence of adverse event? (Very urgent/Moderately urgent/Not urgent)
  - What potential benefit was associated with the management of the illness which led to the Adverse Event? (Life saving/Curing/Life prolonging/Symptom relief/Palliation//No potential benefit)
  - What was the chance of benefit associated with the management of the illness which led to the Adverse Event? (High/Moderate/Low/Not applicable)
  - What was the risk of an adverse event related to the management ? (High/Moderate/Low/Not applicable)
  - Is the injury/complication a recognized complication? (No/Yes/Not applicable)
  - What percentage of patients like this would be expected to have this complication? (Unable to determine/Not applicable/<1%/1-9%/10-24%/>=25%)
  - On reflection, would a reasonable doctor or health professional repeat this health care management strategy again? (Definitely/Probably/Probably not/Definitely not
  - Was there a comment in the medical records indicating a need for follow-up as a result of this Adverse Event? (select all that apply) (No/Counselling/Psychiatric/Rehabilitation/Routine clinical/Other/UTD)
  - Did the patient have any follow-up as a result of this Adverse Event? (No/Counselling/Psychiatric/ Rehabilitation/Routine clinical/Other/UTD)

**Please indicate to what extent there are indications that the event was preventable:**

- - - 1. 1. (Virtually) no evidence for preventability
      2. 2. Slight to modest evidence of preventability
      3. 3. Preventability not quite likely (less than 50/50, but 'close call')
      4. 4. Preventability more than likely (more than 50/50, but 'close call')
      5. 5. Strong evidence of preventability
      6. 6. (Virtually) certain evidence of preventability
